# Does Diagnosing Fatty Liver and Chronic Kidney Disease Do More Good Than Harm?

**DOI:** 10.1371/journal.pmed.1001681

**Published:** 2014-07-22

**Authors:** Druin Burch

**Affiliations:** Public Library of Science, Cambridge, United Kingdom

## Abstract

In a linked Perspective, Druin Burch examines the usefulness of screening for risk factors of cardiovascular disease.

*Please see later in the article for the Editors' Summary*

**Linked Research Article**

This Perspective discusses the following new study published in *PLOS Medicine*:

Musso G, Gambino R, Tabibian JH, Ekstedt M, Kechagias S, et al. (2014) Association of Non-alcoholic Fatty Liver Disease with Chronic Kidney Disease: A Systematic Review and Meta-analysis. PLoS Med 11(7): e1001680. doi:10.1371/journal.pmed.1001680

In a systematic review and meta-analysis, Giovanni Musso and colleagues examine the association between non-alcoholic fatty liver disease and chronic kidney disease.

In this week's issue of *PLOS Medicine*, Musso and colleagues report that two conditions, both occupying increasing amounts of medical attention, may be linked [1]. Their paper demonstrates that non-alcoholic fatty liver disease (NAFLD) is associated with chronic kidney disease (CKD) even after adjustment for major shared risk factors. “Future research,” they write, “should evaluate strategies and interventions to prevent renal disease progression in individuals with NAFLD.” It's a conclusion that is both reasonable and potentially helpful.

Prevalence of NAFLD is estimated at 20% to 40% in Western populations, rising to 75% in those with obesity or diabetes and 90% or more amongst individuals who are morbidly obese. Estimates of NAFLD prevalence in middle-income countries are lower, at 15% to 30%, but are likely to rise with affluence and body weight [2]. Obesity is on course to make fatty liver disease the chief cause of chronic liver disease [3]. NAFLD is a risk factor for cirrhosis but also for the more prevalent problem of cardiovascular harm. It is not inexorable [4]. No intervention has been shown to improve hard clinical outcomes but weight loss and cardiovascular risk reduction are beneficial in themselves and can help normalise histology and liver enzymes [5].

CKD emerged in its modern form at the turn of the century, in papers from 1999 [6] and 2002 [7], which aimed to bring prognostic clarity and aid research and clinical approaches to the problem. CKD also represents a risk factor for specific organ failure and for cardiovascular harm. It too is common, estimated to affect 10% of the global population [8]. Angiotensin converting enzyme inhibitors appear to reduce progression in those with proteinuric CKD but the mainstay of management is once again cardiovascular risk reduction.

CKD and NAFLD represent thoughtful ways of describing deterioration in renal and hepatic health. They help stratify risk and predict future disease burden. They provide tools by which groups can be identified who might best be studied to determine the impact of interventions. Both are also subject to criticism. The most worrying criticisms are not of accuracy but misuse. Critics of CKD have called it a system for “mislabelling large segments of a relatively healthy population as diseased based on innocuous laboratory findings” [9], pointing out that the majority of those with CKD do not progress to end-stage renal failure and do not have complications requiring separate treatment. Most people with CKD do not feel ill, nor have they moved from a state in which their cardiovascular risk was unimportant, nor does their diagnosis mean they gain from new therapeutic options. “For CKD to be a useful concept,” these critics have said, “it requires more than an unsurprising association with cardiovascular disease.” [9].

The production of cardiovascular risk factors can seem an industry oriented more toward the benefit of academic careers than of patients. Fatty liver disease has been caricatured by this author as analogous to “fatty elbow disease” [10]—to a construct that can be coined, found to be prevalent and associated with cardiovascular risk, declared an important global health priority, and yet that is really the creation of an unnecessary disease label. Such a caricature overlooks the utility of fatty liver disease as a research tool but for fatty liver disease to be a useful clinical concept it too requires more than an unsurprising association with cardiovascular disease, or a link with progression to organ failure for which we possess no extra therapeutic interventions [11].

To tell large numbers of people they have a disease, especially when they have no symptoms, is not trivial. Musso and colleagues note “most patients with CKD die from CVD before any renal replacement therapy is initiated” [1]. They cite support for the idea that those with CKD benefit from referral, but the situation, as the paper they reference explains, is unclear. “Key areas of uncertainty [are] identified around the natural history of people with CKD,” it states, pointing out we are uncertain of many aspects of the natural history of CKD as well as of the effectiveness of specific drugs and models of care [12].

There seems no large subset of the population for whom attention to diet, exercise, and salt reduction are not already indicated. Widespread drug treatment of asymptomatic hypertension has long been the norm and statins for those at low levels of cardiovascular risk are now being advocated. These approaches have attracted controversy [13] despite being based on relatively high quality evidence. CKD and NAFLD have not, in contrast, been shown to newly identify those at risk in a way that helps improve outcomes—and the room for them to do so is shrinking. The vast majority of heart attacks occur in those who have already acquired a major risk factor [14]. Under the 2013 American College of Cardiology-American Heart Association (ACC-AHA) guidelines, half of all Americans over the age of 40 qualify for statins [15], and it is interesting to reflect that this now rarely implies disease, the suggestion of treating dyslipidaemia having been shed in favour of accepting the target is cardiovascular risk. Musso and colleagues list the practical implications of their study as indicating the need for lifestyle interventions, the pursuit of smoking cessation, the use of statins and angiotensin receptor blockers, and the assessment against hard endpoints of drug strategies aimed at preventing progression of CKD and NAFLD and their combination [1]. It is the latter proposal that seems novel. An additional possible implication of their work would be the examination of statins in those with raised liver transaminases, a group often excluded from existing trials.

Metabolic syndrome might be described as the natural history of affluent life. The finding of a link between its manifestations in liver and kidney disease, apparently independent of other major cardiovascular risk factors, deserves further examination. The difficulty of fully controlling for confounders is substantial, and what seems of immense importance can fade when analysed in the light of further evidence: even body mass index and waist circumference add little to cardiovascular risk estimates based on diabetes, systolic blood pressure, and lipids. [16]. In situations when it becomes difficult to talk properly of patients and diseases—when there is neither pathos, suffering, nor dis-ease—a strong evidence base is needed to justify intervention. If we bring insufficient health benefits to outweigh the financial and emotional costs of disease labels and clinical activities we are not engaged in medicine but medicalisation. It is odd that the standard of evidence we demand for introducing new clinical approaches falls so far behind that we require of new drugs.

CKD and NAFLD are concepts that can be used or mis-used and their hazards as well as their opportunities merit attention. We are short neither of cardiovascular risk nor cardiovascular risk factors and determining how best to understand and make use of those we possess is not straightforward. Outside of research settings it is essential that the constructs by which we conceive of human frailty do more good than harm, and certainly more good than the notional example of fatty elbow disease.

## Abbreviations

CKD: chronic kidney disease
NAFLD: non-alcoholic fatty liver disease
